# Evaluation of End-User Satisfaction Among Employees Participating in a Web-based Health Risk Assessment With Tailored Feedback

**DOI:** 10.2196/jmir.2067

**Published:** 2012-10-30

**Authors:** Sandra Vosbergen, Eva K Laan, Ersen B Colkesen, Maurice AJ Niessen, Roderik A Kraaijenhagen, Marie-Louise Essink-Bot, Niels Peek

**Affiliations:** ^1^Academic Medical CenterDepartment of Medical InformaticsUniversity of AmsterdamAmsterdamNetherlands; ^2^Academic Medical CenterDepartment of Public HealthUniversity of AmsterdamAmsterdamNetherlands; ^3^NDDO Institute for Prevention and Early Diagnostics (NIPED)AmsterdamNetherlands; ^4^Academic Medical CenterDepartment of CardiologyUniversity of AmsterdamAmsterdamNetherlands

**Keywords:** eHealth, satisfaction, health risk assessment, mixed methods, health behavior

## Abstract

**Background:**

Web technology is increasingly being used to provide individuals with health risk assessments (HRAs) with tailored feedback. End-user satisfaction is an important determinant of the potential impact of HRAs, as this influences program attrition and adherence to behavioral advice.

**Objective:**

The aim of this study was to evaluate end-user satisfaction with a web-based HRA with tailored feedback applied in worksite settings, using mixed (quantitative and qualitative) methods.

**Methods:**

Employees of seven companies in the Netherlands participated in a commercial, web-based, HRA with tailored feedback. The HRA consisted of four components: 1) a health and lifestyle assessment questionnaire, 2) a biometric evaluation, 3) a laboratory evaluation, and 4) tailored feedback consisting of a personal health risk profile and lifestyle behavior advice communicated through a web portal. HRA respondents received an evaluation questionnaire after six weeks. Satisfaction with different parts of the HRA was measured on 5-point Likert scales. A free-text field provided the opportunity to make additional comments.

**Results:**

In total, 2289 employees participated in the HRA program, of which 637 (27.8%) completed the evaluation questionnaire. Quantitative analysis showed that 85.6% of the respondents evaluated the overall HRA positively. The free-text field was filled in by 29.7 % of the respondents (189 out of 637), who made 315 separate remarks. Qualitative evaluation of these data showed that these respondents made critical remarks. Respondents felt restricted by the answer categories of the health and lifestyle assessment questionnaire, which resulted in the feeling that the corresponding feedback could be inadequate. Some respondents perceived the personal risk profile as unnecessarily alarming or suggested providing more explanations, reference values, and a justification of the behavioral advice given. Respondents also requested the opportunity to discuss the feedback with a health professional.

**Conclusions:**

Most people were satisfied with the web-based HRA with tailored feedback. Sources of dissatisfaction were limited opportunities for providing additional health information outside of the predefined health and lifestyle assessment questionnaire and insufficient transparency on the generation of the feedback. Information regarding the aim and content of the HRA should be clear and accurate to prevent unrealistic expectations among end-users. Involving trusted health professionals in the implementation of web-based HRAs may enhance the use of and confidence in the HRA.

## Introduction

Health risk assessments (HRAs) with feedback are commonly used instruments for worksite health promotion [1]. HRAs with feedback screen for risk factors for chronic diseases and provide respondents with information about their health risks, current lifestyle behavior, and opportunities for improving their health. Several studies have already shown promising results for HRAs implemented at the worksite: they might reduce employees’ health risks and improve their lifestyles [1]. This is also beneficial for the employer, as employees with healthy lifestyles are generally absent from work less often and are more productive than employees with unhealthy lifestyles [2-4].

Nowadays, HRAs are increasingly offered as web-based applications. The use of computer technology and email provides the opportunity to reach large groups of individuals and to deliver individually tailored feedback [5]. Following Kreuter et al [6], we define tailoring as “any combination of information or change strategies intended to reach one specific person, based on characteristics that are unique to that person, related to the outcome of interest, and have been derived from an individual assessment.” Within the worksite setting, emails can reach diverse employee populations [7]. Emails and web pages offer a natural way of reaching employees, as it fits into their daily work routine. Employees are also able to use computer programs in private, for example, at home, and at a time that suits them [8].

Although the potential of web-based HRAs with tailored feedback at the worksite is clear, few evaluations take into account satisfaction from an end-user perspective. To date, studies that did take this into account were either focused on eHealth approaches (eg, telemedicine) [9,10] other than HRAs, studied satisfaction only as an effectiveness measure without studying factors that affect end-user satisfaction [11-13], or evaluated non−web-based health promotion programs [11,13,14]. However, end-user satisfaction is shown to be positively related to compliance to medical regimes in primary health care [15] and with initiation of health behavior change after participating in the HRA studied here [16]. Respondents who were more satisfied with the HRA were nearly three times more likely to initiate lifestyle changes after participation [16]. Yet to our knowledge, no studies have comprehensively studied satisfaction and the factors affecting it. Furthermore, more extensive evaluation of workplace health promotion programs is needed as the most effective strategies for these services have not yet been determined [17]. Evaluating the factors described within the conceptual framework of Wixom and Todd [18] can give insight into how web-based HRAs with tailored feedback are used and provide opportunities for improvement. By evaluating HRAs after implementing them in the proposed setting on a voluntary basis without remuneration, real-life personal experiences with the HRA could be assessed and used to inform the design process.

### Objectives

The aim of this study was to evaluate end-user satisfaction with a web-based HRA with tailored feedback applied in worksite settings, using mixed (quantitative and qualitative) methods. Mixed methods can provide insights that may be missed when only a single research modality is used [19]. Satisfaction with different components of the HRA, and determinants affecting this satisfaction were evaluated both quantitatively and qualitatively. Qualitative methods were also used to determine opportunities for improvement.

## Methods

### Design and Study Population

This study was conducted between September 2007 and December 2008. Seven companies in the Netherlands invited their employees to participate in a commercial, web-based HRA with tailored feedback. The HRA was part of the companies’ corporate health-management strategy. Two companies used an age-based inclusion criterion for participation in the HRA (35 years and older and 45 years and older, respectively). The other companies did not use inclusion criteria, and all employees were invited to participate. The companies’ human resources departments sent the invitations for participation in the HRA by email. Employees were informed that participation was voluntary and free of charge, that all personal data would be treated confidentially, and that no individual results would be shared with their employer or with any other third parties. In case of no response to the invitation email, a single reminder was sent two weeks after the initial invitation.

Six weeks after participation in the HRA, participants received an invitation by email for an electronic evaluation questionnaire. This electronic evaluation questionnaire was not part of the HRA but was sent for research purposes.

### Intervention

The HRA consisted of four components: 1) an electronic health and lifestyle assessment questionnaire, 2) a biometric evaluation, 3) a laboratory evaluation, and 4) tailored feedback consisting of a personal health risk profile and lifestyle behavioral advice communicated through a web portal.

The health and lifestyle assessment questionnaire covered sociodemographic variables, family and personal medical history, health complaints, psychological functioning, and lifestyle behavior (physical activity, smoking behavior, alcohol consumption, nutrition intake, and stress). Furthermore, participants were asked to assess their own lifestyle on a scale from 1 to 10 and their health perception on a 5-point Likert scale (very good, good, neither good nor poor, poor, very poor). More details on the health and lifestyle assessment questionnaire can be found in Laan et al [20].

The biometric evaluation consisted of measurements of weight, height, waist circumference, and blood pressure taken by certified health professionals. Furthermore, samples of blood, urine, and feces were taken for lab analyses of total cholesterol, low-density lipoprotein (LDL) cholesterol, high-density lipoprotein (HDL) cholesterol, triglycerides, glucose, creatinine, and urinary albumin-to-creatinine ratio. To collect and analyze feces samples and provide feedback on the results, the developer of the HRA (NDDO Institute for Prevention and Early Diagnostics (NIPED)) received permission to screen for colorectal cancer under Dutch law, provided by the Dutch Ministry of Health, Welfare, and Sport.

**Figure 1:**
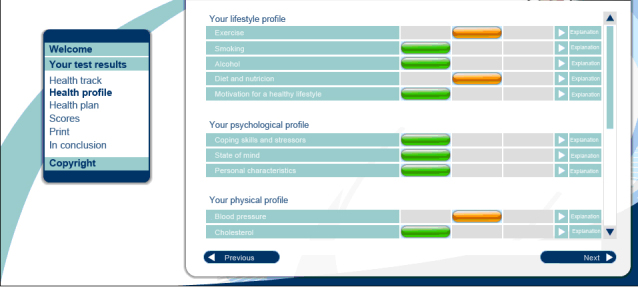
Screenshot of the personal health risk profile page.

**Figure 2:**
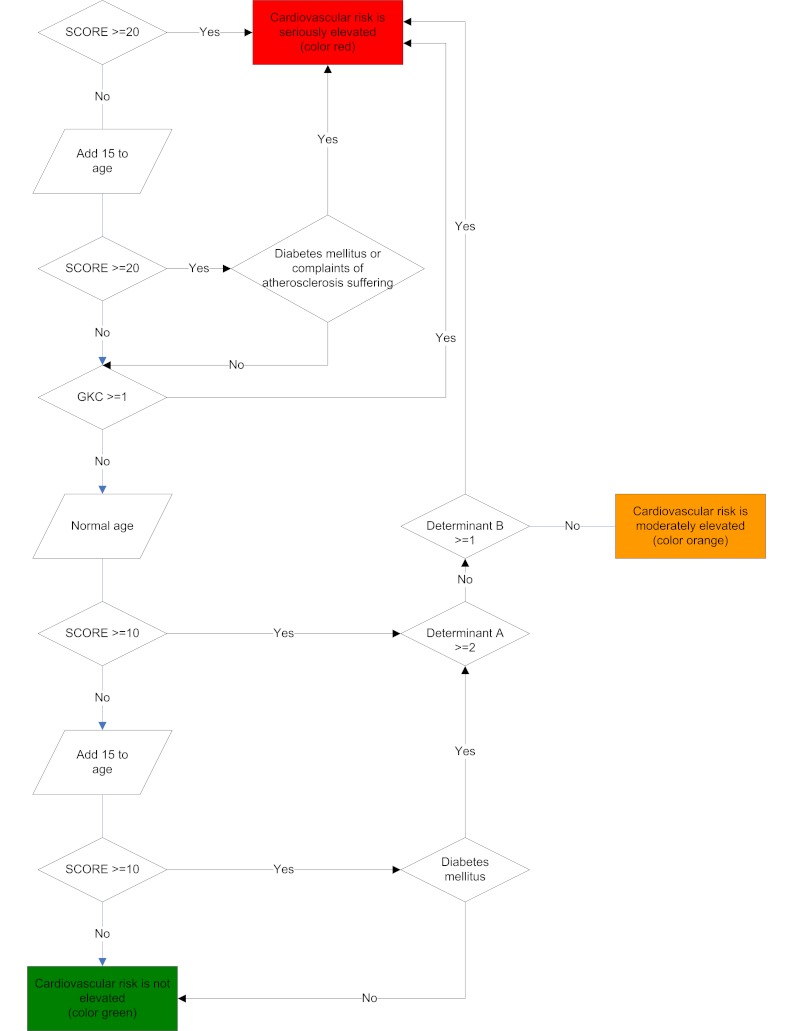
Algorithm for cardiometabolic health risk.

After completing all HRA components, the information collected from both the biometric evaluation and the health and lifestyle questionnaire was processed by computer algorithms to compute the tailored feedback. This feedback was provided to the user immediately after completion of the questionnaire, the biometric evaluation, and after the laboratory provided feedback on the results of blood, urine, and feces. When the tailored feedback was available, the user received an email. The feedback was divided into five health-related domains (behavioral, psychological, physical, personal medical history/familiar risk, and work-related). For each of these domains, 1) a three-color system was used to explain the health risk (green: normal risk; orange: moderately elevated risk; red: seriously elevated risk), and 2) the threats associated with elevated risk (orange and red categories) and the potential gains of taking preventive action were explained. A compass metaphor was used to summarize overall health risk, with the categories “on track (color green)”, “slightly off-track (color light orange)”, “moderately off-track (color dark orange)”, and “seriously off-track (color red)”. In the remainder of this article we will refer to those categories by their colors. All risk calculations were based on prevailing practice guidelines, including the European and Dutch guidelines for cardiovascular risk management [21,22]. The feedback concluded with comprehensive suggestions of actions the participant could take. All options suggested trusted external parties the participant could go to for support for the action they might want to take. These suggestions were made based on their expressed preferences (such as for guided versus non-guided interventions, actions in groups or on their own, and actions away from or at home), and differentiated between the participant’s stage of motivation for lifestyle changes (transtheoretical model of health behavior change [23]). In case of seriously elevated health risks, the feedback included a referral to a general practitioner (GP) for further medical evaluation and treatment if necessary. For all participants, a 30-minute health counseling visit with the program physician was available on request. An example of feedback provided by the system is shown in Multimedia Appendix 1 (see also Figure 1), and a more extensive description of how the feedback is generated can be found in Multimedia Appendix 2 (see also Figure 2).

### Measurements

Six weeks after receiving the feedback, participants received a two-part electronic evaluation questionnaire. The first part assessed satisfaction with the HRA, and the second part assessed initiation of health behavior change after participation in the HRA. The questionnaire was sent using an email survey program, with a single reminder after one week. The findings on initiation of health behavior change have been published elsewhere [16].

The part of the evaluation questionnaire related to satisfaction with the HRA—which is the focus of this study—consisted of 10 items. Respondents were asked to appraise (1) pre-program information and communication about the HRA, (2) the registration procedure, (3) the electronic health and lifestyle assessment questionnaire, (4) the planning of the biometric evaluation visit, (5) the biometric evaluation visit, (6) the comprehensibility of the provided feedback, and (7) the optional health counseling visit with the program physician. These items refer to successive steps in the HRA procedure, each of which has to be completed before the next one is started; there is no overlap. Satisfaction with each step was measured on a 5-point ordinal ranking scale (excellent, very good, good, average, poor). In addition, overall satisfaction with the HRA was measured with two questions: (8) “How do you assess your participationin the HRA in general?” (measured on a 5-point ordinal ranking scale: excellent, very good, good, average, poor) and (9) “Would you recommend the HRA to others?” (measured on a 5-point agreement scale: definitely would, probably would, maybe, probably not, definitely not). Finally, a free-text field was available for additional comments. The corresponding instruction read “It’s possible that some things were not contained in the above questions, or that you weren’t able to express these things as you would have liked. If this is the case, please enter them below.”

#### Data Analyses

##### Quantitative Analysis of Satisfaction With the HRA

Three items of the questionnaire were not included in the data analyses: pre-program information, the registration procedure, and the planning of the biometric evaluation visit. The first two markedly differed between companies, and no remarks in the free-text field on these components of the HRA were made. The planning of the biometric evaluation visit was excluded because this practical aspect had no direct link with the web-based component of the HRA.

Descriptive statistics were performed on all data to examine population characteristics and satisfaction with the HRA. For satisfaction with the different components of the HRA and the overall satisfaction with the HRA, the response options were dichotomized into “positive judgment” (excellent, very good, good) and “negative judgment” (average, poor) because of unbalanced ranking scales. Response bias to the evaluation questionnaire was checked by comparing respondents and non-respondents on demographic characteristics, educational level, health perception, self-rated lifestyle, body mass index, physical activity, fruit and vegetable intake, smoking behavior, alcohol consumption, and overall health risk. Response bias to the free-text field was checked by comparing respondents who made remarks in the free-text field and respondents who did not on demographic characteristics and satisfaction. For both response bias analyses, *t* test for continuous outcomes, Chi-square tests for dichotomous outcomes, and Mann-Whitney (for two groups) and Kruskal-Wallis tests (for more than two groups) for ordinal outcomes were used. The Statistical Package for the Social Sciences (SPSS) version 18 was used to perform statistical analyses.

##### Qualitative Analysis of Satisfaction With the HRA

To analyze the textual remarks, a codebook was developed based on concepts from the user satisfaction and technology acceptance literature [18]. User satisfaction and technology acceptance theories are used to evaluate users’ perceptions about information systems to predict actual usage of these systems. The theories use a characteristics-based approach, with the potential end-user’s attitude towards a system as pivotal construct. There are different theories that take varying system characteristics into account. Because we aimed to evaluate the HRA in the broadest sense, we included in our codebook all concepts as described in Wixom and Todd, 2005 Table 1, p. 88) [18]. At the start of the analysis, all domains and concepts were adopted directly from the table. Interpretations of both were specified to the HRA evaluated in the current study. Domains, concepts, and interpretations are listed in Multimedia Appendix 3.

Two researchers (SV and EL) independently categorized the remarks according to three different topic schemes: 1) the component of the HRA addressed (pre-program information and communication about the HRA, the registration procedure, the electronic health and lifestyle assessment questionnaire, the planning of the biometric evaluation visit and the visit itself, the feedback provided by the system, counseling visit with the program physician, or the HRA in general), 2) domain and concept from the codebook (see Multimedia Appendix 3), and 3) whether the remark was positive, negative, or neutral. Considering that remarks sometimes referred to different components of the HRA, they were divided into shorter remarks when necessary, one for each component of the HRA addressed. If these shorter remarks covered different concepts from the codebook, they were again divided into even smaller remarks. If in the original codebook a remark did not pertain to any concept of a domain, we looked for a concept from another domain and copied that between the domains. For example, although “feelings of control” was originally described only in the domain “outcome expectations,” in our analysis this concept was also needed in the domain “information quality,” so we added this concept here. If no suitable concept was found, a new one was added. Changes to the codebook made during the analyses are also described in Multimedia Appendix 3.

During four subsequent meetings, we tried to reach consensus about the categorization by comparing and discussing this. If consensus could not be reached, a third researcher (NP) was brought in to resolve the disagreement.

##### Analysis of Determinants

Univariate logistic regression analyses were used to assess the associations between respondent characteristics (gender, age, educational level, health perception, self-rated lifestyle, and overall health risk) and satisfaction with each of the program components (health and lifestyle assessment questionnaire, visit for biometric evaluation, comprehensibility of feedback, and health counseling visit with program physician) as well as overall satisfaction with the HRA and intention to recommend the HRA to others. Prior to the analyses, age was categorized into “younger than 50 years” and “50 years and older.” Because a majority of the respondents rated their own lifestyles with the number 7, this variable was categorized into three categories: 1−6, 7, and 8−10. Baseline data also showed that the distribution of health perception was unbalanced; only a few respondents assessed their health perception as “neither good nor poor”, “poor”, or “very poor”. Therefore, health perception was categorized into “very good”, “good”, and “poor” (comprising the categories “neither good nor poor”, “poor”, and “very poor”). Finally, the response scale for intention to recommend the HRA to others was dichotomized into “positive intention” (definitely would, probably would) and “negative intention” (maybe, probably not, definitely not). Overall, 36 univariate logistic regression analyses were performed, the results of which were quantified as odds ratios.

If a respondent characteristic was found to be significantly associated with a judgment of satisfaction with (components of) the HRA, the remarks that were entered into the free text field were subsequently searched for explanations. This was done by contrasting the remarks of different groups of respondents, as defined by the characteristic. For instance, explanations of significant associations with age were searched for by contrasting the remarks made by respondents younger than 50 years with those made by respondents of 50 years and older.

## Results

### Study Population

A total of 6790 employees were invited to participate in the HRA. Of those, 2289 (33.7%) completed all components of the HRA measurements and thus received feedback by the system. 183 out of 2472 (7.4%) dropped out after they started with the HRA. The response to the evaluation questionnaire was 27.8% (637/2289). Table 1 shows the characteristics of the respondents who completed the evaluation questionnaire.

**Table 1:** Characteristics of respondents to the evaluation questionnaire (N=637).

Variable	Value
Male sex, N (%)	386 (60.6)
Age, mean ± SD	46.49 ± 8.76
≥ 50, N (%)	230 (36.1)
Education level	
low, N (%)	139 (21.8)
moderate, N (%)	191 (30.0)
high, N (%)	307 (48.2)
Health perception	
very good, N (%)	116 (18.2)
good, N (%)	415 (65.1)
not good, not bad, N (%)	102 (16.0)
bad, N (%)	4 (1)
very bad, N (%)	0 (0)
Self-rated lifestyle (1−10), mean ± SD	7.18 ± 0.98
1−6, N (%)	119 (18.7)
7, N (%)	275 (43.2)
8−10, N (%)	243 (38.1)
Body Mass Index, mean ± SD	28.09 ± 3.77
Physical activity (min/week), median [interquartile interval]	165 [60−290]
Fruit (pieces/day), mean ± SD	1.76 ± 0.43
Vegetables (g/day), mean ± SD	1.56 ± 0.50
Currently smoking, N (%)	146 (22.9)
Alcohol consumption	
less than 1 unit/week, N(%)	160 (25.1)
1–7 units/week, N(%)	268 (42.1)
8–14 units/week, N(%)	126 (19.8)
15–21 units/week, N(%)	54 (9)
22 or more units/week, N (%)	29 (5)

 There were no significant differences between respondents and non-respondents to the evaluation questionnaire, except for age. On average, respondents to the evaluation questionnaire were one year older (mean ± SD: 46.5 ± 8.8 years) than those who did not respond to the evaluation questionnaire (mean ± SD: 45.5 ± 8.5 years) (*P* =.01) (data not shown). Except for age and physical activity, there were also no significant differences between respondents who made remarks in the free-text field (either a negative, positive, or neutral remark) compared to those who did not. Respondents who made remarks in the free-text field were nearly two years older (mean ± SD: 47.8 ± 8.1 years) than those who did not make remarks (mean ± SD: 45.9 ± 9.0 years) (*P* =.01) (data not shown). Respondents who made remarks in the free-text field were less physically active (median [interquartile interval (IQI)]: 150 (50–270) min/week) compared to those who did not made remarks (median [IQI]: 170 (60–300) min/week) (*P*<.01) (data not shown).

### Satisfaction With the HRA

Table 2 shows the satisfaction rankings of the respondents to the evaluation questionnaire. Additional analyses showed that fewer respondents who made one or more remarks in the free-text field were satisfied overall with the HRA (134 out of 189, 70.9%) compared to those who did not make remarks (411 out of 448, 91.7%) (*P<*.01) (data not shown). Similar figures were found for the health and lifestyle assessment questionnaire, for the visit for biometric evaluation, for the comprehensibility of the feedback, and for the intention to recommend the HRA to others. However, more respondents who made remarks judged the health counseling visit with the program physician as positive (40 out of 69, 58.0%), compared to those who did not make remarks (130 out of 157, 29.0%) (*P<*.01).

**Table 2:** Numbers and percentages of respondents that judged positively and negatively about (components of) the HRA (N=637).

Judgments of HRA components	Number (percentage)
Health and lifestyle assessment questionnaire	
positive, N (%)	557 (87.4)
negative, N (%)	80 (13)
Biometric evaluation visit	
positive, N (%)	550 (86.3)
negative, N (%)	82 (13)
no answer	5 (1)
Comprehensibility of the feedback	
positive, N (%)	515 (80.8)
negative, N (%)	119 (18.7)
no answer	3 (1)
Health counseling visit with the program physician	
positive, N (%)	170 (75.2ª)
negative, N (%)	56 (25ª)
Overall satisfaction with the HRA	
positive, N (%)	545 (85.6)
negative, N (%)	92 (14)
Intention to recommend the HRA to others	
definitely would, N (%)	274 (43.0)
probably would, N (%)	198 (31.1)
maybe, N (%)	107 (16.8)
probably not, N (%)	44 (7)
definitely not, N (%)	14 (2)

^a^ Percentage based on those respondents who actually visited the program physician (N=226).

#### End-users’ Remarks About the HRA

The free-text field at the end of the questionnaire was used to analyze the data qualitatively; 189 out of the 637 respondents (29.7%) filled in this field. One hundred and twelve respondents made one remark, 45 respondents made two remarks, 20 respondents made three remarks, and 12 respondents made four or more remarks. In total, 315 separate remarks were made. Of those 315 remarks, 33 (10%) were positive, 249 (78.3%) were negative, and 33 (10%) were neutral.

##### Assessment Phase

80 remarks were made by 70 respondents about the assessment phase. Of those remarks, 3 (4%) were positive, 71 (88.8%) were negative, and 6 (8%) were neutral.

 Most remarks regarding the health and lifestyle assessment questionnaire were about limitations in opportunities for expressing individual details. There were respondents who felt limited in their ability to enter all the information about their health or lifestyle that they considered relevant (eg, existing health problems like food allergies and back pain). It was also mentioned that the questionnaire was insufficiently tailored to respondents’ personal situation (eg, a lack of gender-specific questions). This resulted in the perception that the assessed personal health risk profile did not adequately represent respondents’ actual health status.

I realize you want the phrasing of the questions to be as clear as possible. In a number of cases, the answers are oversimplified. The actual situation is sometimes far removed from the possible answers, and consequently the results also give a different (more negative) picture.

Male, age 52

Because of this perceived restriction, these respondents indicated they preferred speaking to a health professional after completing the health and lifestyle assessment questionnaire or expected to be able to provide additional information or ask questions during the visit for biometric evaluation. However, this visit was facilitated by an external organization that had only limited information about the HRA. Their only task was to obtain the measurements. This affected respondents’ satisfaction with the biometric evaluation.

I thought the biometric evaluation visit was quite basic. All of the procedures were carried out in a rather impersonal way and at breakneck speed. It felt a little bit like a production line.

Female, age 38

To improve the system with regard to the perceived restrictions, respondents suggested adding a text field to the questionnaire where they could enter additional information or ask questions.

##### Personal Risk Profile and Lifestyle Behavior Advice

83 remarks were made by 68 respondents about the personal risk profile and lifestyle behavior advice. Of those remarks, 1 (1%) was positive, 75 (90.4%) were negative, and 7 (8%) were neutral.

The personal risk profile was criticized because of the format of the information provided. There were respondents who perceived the risk profile as unnecessarily alarming.

The way in which the results were presented meant I didn’t sleep very well for a number of nights, even though afterwards the company doctor and my GP said there was no reason to take any further steps.

Female, age 40

Feelings of anxiety and lack of confidence in the feedback provided by the system often resulted in a second opinion by the respondent’s own GP.

Others criticized the lifestyle behavior advice for being too complex, and sometimes for being too trivial (eg, respondents felt the advice included complex medical terminology or perceived the advice as simple as it told them to exercise more). Furthermore, there were respondents who expected more guidance in the execution of the lifestyle behavior advice.

I had the impression I’d get more help. But the answers I got were things I already knew. For instance, that I’d like help in trying to lose weight. It just said I could contact my GP.

Male, age 36

Finally, some respondents argued that the suggestions for taking action were easy to ignore.

Because the communication is all on paper, it’s easy to ignore any recommendations.

Male, age 55

The HRA was also criticized because the relation between information entered and feedback provided by the system was not always clear to respondents.

I’d like to see a clearer connection between output and input. For example, if there’s a recommendation to eat more dairy products, does this come out of the tests or from the questionnaire? I think it’s important that the client knows what a recommendation is based on.

Male, age 40

Especially when the feedback contained unexpected information, respondents wanted to review their answers on the questionnaire and have the possibility to link these to the provided feedback—but the HRA did not support this. Perceived contradictions in the feedback sometimes led to irritation.

What stands out for me is that even though I didn’t get any red scores, I’m seriously off-track and was referred to the prevention consultant. This doesn’t add up.

Male, age 47

Also, some respondents mentioned a lack of confidence in the feedback provided because it was contrary to their expectations.

Many respondents who made a remark mentioned that the personal risk profile did not provide threshold values, especially with regard to the biometric measures. As a result, some of these respondents indicated they did not know how to interpret the feedback.

When the results are shown, there’s not enough mention of reference materials … the result is 4, but on which scale? … What’s good and what isn’t, and when or at what score should I be concerned?

Male, age 42

##### Applying the Advice in Practice

39 remarks were made by 28 respondents about applying the advice in practice. Of those remarks, 2 (5%) were positive, 34 (87.2%) were negative, and 3 (8%) were neutral.

Despite the fact that a health counseling visit was available to every respondent, there were respondents who criticized the HRA for the absence of such a visit. This was likely due to a communication problem. Other respondents said that web-based feedback was rather impersonal compared to feedback provided by a health care professional.

Respondents made remarks about their experiences after receiving the feedback. Most respondents who went to their GP either because the provided feedback included referral to their GP or because they wanted a second opinion, underwent another biometric evaluation. Reasons they reported for this were that the GP perceived the feedback as being unclear and unusable, disagreed with the threshold values that were used for risk stratification, or claimed that certain measures were not performed correctly.

#### Overall Satisfaction With the HRA

104 remarks were made by 84 respondents about overall satisfaction with the HRA. Of those remarks, 27 (26.0%) were positive, 64 (61.5%) were negative, and 13 (12.5%) were neutral.

Respondents who made remarks in the free-text field differed in their opinions about the HRA’s usefulness. There were respondents who considered the HRA to be superficial, commercial, of limited value, and/or not useful without a long-term trajectory:

To me, it was an automated, watered-down version of my own input. I reported that I had high blood pressure and—surprise—the program affirms this. I say I sometimes don’t eat two servings of fruit a day. And presto, the program says I should eat more fruit. I indicate I’m going to exercise more, but the program isn’t interested in this because I’ve already sufficiently answered the questions on exercise.

Male, age 53

Furthermore, respondents indicated a preference for a more personal approach.

During a face to face talk, you could have given a lot more information and clarified things and also have had a more thorough physical examination.

Female, age 42

For a few respondents, participating in the HRA was very useful because it warned them of serious health problems such as hypertension and cancer. They underwent appropriate medical interventions and were grateful they participated, something that was illustrated by the following respondent:

A polyp has been removed from my intestines on two different occasions. According to the specialist, one of these would certainly have become malignant.

Male, age 46

Positive remarks were also made by respondents who received confirmation they were in good health, respondents who perceived the HRA as being a good point of reference for their health, and those who considered prevention in general to be useful, as illustrated by the following quote:

I think it’s a good idea to have one’s weight checked, blood tested for cholesterol, and so forth. Everyone should. Sometimes diseases, abnormalities, come to light (not now, in my case), and then it makes a big difference if this is caught in time. As far as I’m concerned, this can be done every year or every two years.

Female, age 40

The biometric evaluation was perceived by some respondents as the most useful component of the HRA.

#### Determinants Affecting Satisfaction With the HRA

Table 3 shows the associations between respondent characteristics and their satisfaction with (components of) the HRA. There were significant differences between men and women in the judgment of the health and lifestyle assessment questionnaire. Women were almost twice more likely to be dissatisfied than men (OR=0.54, *P*=.01). Qualitative analysis showed that there were no differences in the contents of remarks made by men and women in the free-text field though.

Furthermore, significant differences were found in the judgment of the comprehensibility of the feedback amongst groups with different risk profiles. None of the respondents with a green overall risk profile evaluated the comprehensibility of the feedback negatively. Those who received a light orange (OR=1.93, *P*=.02) and dark orange (OR=1.87, *P*<.01) risk profile were more likely to judge the comprehensibility of the feedback positive than those who received a red risk profile. Also, the intention to recommend the HRA varied by risk profile; those who received a green risk profile were much more likely to have the intention to recommend the HRA to others than those who received a red profile (OR=4.52, *P*=.02).

Comparisons of the remarks made by respondents with different risk profiles showed that respondents with a red risk profile perceived more difficulties with applying the lifestyle behavior advice and reported anxiety as a result of the health risk profile than respondents with other risk profiles. For instance, remarks regarding the applicability of the lifestyle behavior advice or disagreement with the provided feedback by GPs were made by respondents with a red risk profile. No remarks regarding the provided feedback were made by respondents with a green risk profile.

In general, respondents with a green risk profile made relatively more positive remarks compared to respondents with other risk profiles. Also, their remarks were about practical issues like the service provided or materials used at the biometric evaluation visit and the evaluation questionnaire itself and no issues related to the web-based component of the HRA.

**Table 3:** Associations of positive judgments with respondent characteristics, expressed as odd ratios with 95% confidence intervals (N=637).

	Judgment of the health and lifestyle assessment questionnaire	Judgment of the visit for biometric evaluation	Judgment of the comprehensi-bility of feedback	Judgment of the health counseling visit with the program physician	Overall satisfaction with the HRA	Intention to recommend the HRA to others
Gender						
Male	ref. category	ref. category	ref. category	ref. category	ref. category	ref. category
Female	0.54 [0.34–0.87]^b^	0.79 [0.49–1.26]	0.87 [0.58–1.30]	0.73 [0.40−1.35]	0.74 [0.47–1.16]	0.69 [0.48–0.99]^b^
Age						
< 50	ref. category	ref. category	ref. category	ref. category	ref. category	ref. category
50 and older	0.93 [0.58−1.52]	1.34 [0.82–2.22]	0.91 [0.61–1.38]	1.40 [0.76–2.58]	0.82 [0.52−1.28]	1.18 [0.81−1.71]
Educational level						
Low	ref. category	ref. category	ref. category	ref. category	ref. category	ref. category
Moderate	1.02 [0.55−1.91]	1.03 [0.55−1.90]	1.38 [0.80−2.17]	0.76 [0.36−1.62]	1.45 [0.79–2.68]	1.32 [0.81–2.16]
High	1.40 [0.77–2.53]	1.49 [0.82–2.67]	1.32 [0.81–2.17]	1.47 [0.68–3.16]	1.25 [0.72–2.15]	1.23 [0.79−1.92]
Health perception						
Poor	ref. category	ref. category	ref. category	ref. category	ref. category	ref. category
Good	1.04 [0.56–1.91]	0.65 [0.33−1.29]	1.09 [0.65−1.83]	0.82 [0.39–1.73]	0.76 [0.41–1.42]	0.86 [0.53−1.40]
Very good	2.23 [0.90–5.49]	1.35 [0.54−3.40]	2.38 [1.12–5.06]^b^	1.96 [0.62–6.16]	1.81 [0.75–4.37]	1.64 [0.86–3.14]
Self-rated lifestyle						
1 t/m 6	ref. category	ref. category	ref. category	ref. category	ref. category	ref. category
7	1.14 [0.61–2.13]	0.48 [0.23–1.03]	1.00 [0.58–1.72]	0.739 [0.34−1.61]	0.98 [0.53−1.81]	0.70 [0.43−1.16]
8 t/m 10	1.28 [0.67–2.44]	0.52 [0.24–1.12]	1.10 [0.63–1.94]	1.03 [0.46–2.29]	0.99 [0.53−1.85]	0.96 [0.57−1.61]
Overall health risk						
Green	0.83 [0.29–2.37]	6.20 [0.81–47.2]	N/A^a^	4.14 [0.46 – 34.64]	4.07 [0.93–17.75]	4.52 [1.13–15.37]^b^
Light orange	1.28 [0.60–2.72]	0.93 [0.50–1.73]	1.93 [1.10−1.87]^b^	1.30 [0.56–3.02]	1.15 [0.63–2.08]	0.79 [0.49–1.29]
Dark orange	0.71 [0.40–1.25]	1.27 [0.73–2.19]	1.87 [1.19–2.94]^b^	1.75 [0.88–3.49]	1.62 [0.97–2.72]	1.24 [0.81−1.90]
Red	ref. category	ref. category	ref. category	ref. category	ref. category	ref. category

^a^ Odds-ratio and 95% confidence interval could not be calculated because all respondents with a green overall health risk evaluated the comprehensibility of the feedback positive.

^b^ *P*< .05.

## Discussion

### Main Findings

This study evaluated end-user satisfaction with a web-based HRA with tailored feedback. Quantitative evaluation data showed that most respondents were satisfied with the HRA in general and with its constituent components. Nearly three-quarters (74%) of the respondents indicated that they would definitely recommend the HRA to others or considered doing so. Overall, respondents who were negative about (components of) the HRA also made critical remarks in the free-text field.

Critical remarks about the HRA found in the qualitative analysis related to perceived control over the entered information, confidence in the generated feedback, and embedding of the HRA in the health care system. Several respondents requested more insight into the generation of the feedback. This and the other results of this study suggest that transparency of the underlying computer system is important for confidence in the feedback provided. If the feedback provided by the HRA did not match the respondent’s expectations, they wanted the system to provide convincing arguments for it, based on the information they had entered. Several remarks also indicated that there were respondents who had more confidence in health professionals than in a computer system. Respondents often mentioned that they went to their GP for a second opinion, as they had no confidence in the feedback provided. There were also respondents who requested the opportunity to discuss the feedback with a health professional.

### Relationship to Other Studies

In a systematic review of interventions for worksite health promotion, Soler et al reported two potential adverse effects of HRAs with feedback [1]. First, the feedback provided by the HRA could cause anxiety in participants, and second, there may be false-positive results (ie, the feedback incorrectly indicates there is health problem). Both adverse effects were reported in our study. Some respondents became anxious when the health plan showed alarming results. Consequently, most of them indicated that they went to their GP for a second opinion. The GP often told these respondents there was nothing to worry about. These signals may either have been false alarms or correct warnings that were not recognized by the GPs. In principle, the latter seems more likely as both the risk calculations and feedback given by the HRA followed prevailing Dutch practice guidelines. So probably GPs were sometimes acting against their own guidelines during the visit. We think this can be explained by unfamiliarity of GPs with the HRA and perhaps a lack of transparency for GPs with respect to the decision rules underlying the feedback. Another possible explanation is the fact that GPs in the Netherlands act as “gatekeepers” to the health care system and tend to act reluctantly.

While there are an increasing number of studies on information systems targeted directly at care consumers or patients, there is more extensive literature on the use of information systems by medical professionals. A systematic review of clinical decision support systems for medical professionals by Kawamoto et al showed that clinical practice is more likely to improve if users are provided with actionable recommendations rather than mere assessments [24]. The HRA evaluated in our study does provide such actionable recommendations, which were appreciated as such by the respondents. Yet our findings also indicate that the advice should be suitably adapted to the user’s needs, preferences, and characteristics. Kawamoto et al also found that the effectiveness of decision support systems might potentially benefit if recommendations given by the system are justified by providing the underlying line of reasoning or research evidence [24]. Our findings support that justifying the provided recommendations has added value for the system’s end-users, especially for their confidence in the recommendations.

In a study on an automated health behavior change intervention, Bickmore et al found that relational behaviors (ie, empathy and social dialogue) improved the liking of and the satisfaction, relationship, and desire to continue with the system [25]. There were respondents in our study who indicated they preferred to discuss their feedback with a health professional face to face, which supports Bickmore’s finding that adding relational behaviors to the HRA interface might increase the perceived value of the HRA.

In their qualitative study, Wolff et al found various opportunities and barriers to disease prevention counseling in primary care [26]. The three aspects participants of their study requested most were tailored information, encouragement, and follow-up. The results of our study are in line with these findings: among the respondents, there were requests for more tailoring of information, positive feedback, and in some cases respondents felt the need for a second opinion or another form of professional follow-up after using the HRA.

### Strengths and Limitations

In our study, we evaluated end-user satisfaction with a web-based HRA using both quantitative and qualitative methods. As described in the introduction, using mixed methods can provide additional insights that may be missed when only a single research modality is used [19]. In our case, although respondents were generally satisfied with the HRA, the qualitative data contained predominantly critical remarks. The qualitative data showed important aspects to consider when developing or implementing a web-based HRA. Furthermore, by analyzing respondent’s remarks in the broadest sense, this study gave insight into both aspects related to web-based part of the HRA as well as the implementation of the HRA.

During the categorization of remarks, all domains and most concepts of the framework were used. Only one new concept was added, and some concepts were used in more than one domain or not used at all (see Multimedia Appendix 3). Because no new domains were needed and only a few adjustments were made to the framework, we conclude that the used framework was suitable for analyzing the data of this study.

The present study does have several limitations. A large majority of participants did complete all elements of the HRA; however, 183 out of 2472 (7.4%) participants dropped out before completing the HRA and did not receive an evaluation questionnaire. Furthermore, the response to the evaluation questionnaire was 28%, which is lower than the mean response of 60% to 67% in most satisfaction surveys [27,28]. However, our response is comparable to the response for general email health surveys, which is around 34% [29]. In addition, no indication for selection bias was found, except for age.

Two companies applied an age-based criterion because they believed that HRAs are more beneficial to older employees. Companies without an age-based criterion argued they wanted to give all their employees, regardless of age, the opportunity to participate in the intervention. This might have caused a selection bias. However, complementary analysis showed this bias is small. There is a small difference in the distribution of age between all companies included in this study (age, mean ± SD: 46.5 ± 8.8) and companies without the age-based criterion (age, mean ± SD: 45.9 ± 8.9). For gender, the difference between all companies and those with the age-based criterion was respectively 386 out of 637 (60.6%) and 351 out of 553 (63.5%). Furthermore, this analysis showed that there were no significant differences between companies with and without this age-based criterion on satisfaction, except for satisfaction with the health counseling visit with the program physician (data not shown).

Self-reported data concerning health status should be interpreted with care, as they may be influenced by social factors. In addition, the phrasing of the question of the free-text field might have influenced the number and the subjects of the remarks made by respondents. The free-text field was not a mandatory field, which could explain the low percentage of respondents who actually made remarks. In general, free-text fields take more time and therefore more effort to fill in, which could also explain the low number of remarks. More important is that the free-text field was intended for additional remarks, which could have resulted in a lower number of these. Interpreting the term “additional” as “other than what was mentioned in the evaluation questionnaire” could also result in remarks unrelated to the items contained in the evaluation questionnaire. This could explain the apparent discrepancy between the results found in the quantitative and the qualitative data. Despite the limitations of the phrasing of the free-text field question, we assumed that if respondents had remarks, they would write those down anyway. Subsequently, unbalanced ordinal ranking scales were used in the evaluation questionnaire. An unbalanced scale was used to rank satisfaction with the different components of the HRA program. Therefore we dichotomized the scale into positive (excellent, very good, good) and negative (average, poor) rankings. The item “Intention to recommend the HRA to others” was ranked on a balanced scale.

Several components of the HRA (pre-program information, the registration procedure, and the planning of the biometric evaluation visit) were excluded beforehand from the analysis. Therefore, we cannot draw conclusions about the above described components of the HRA or about the influence of these components on the satisfaction rankings. Furthermore, although we believe the framework we used was suitable for the categorization of our data, some of the remarks of participants did not fully fit the framework. Therefore, extending the framework with, for example, aspects of the “Diffusion of innovations” theory [30] might have put our findings in another perspective.

Finally, the quantitative analysis showed that critical remarks in the free-text field were made by respondents who were less satisfied with the HRA. This suggests that those who are less satisfied are more inclined to make remarks, which explains the predominantly negative remarks. Still, we believe that these remarks should be taken seriously and that they could support us in improving future HRAs and increase the utilization of HRAs.

### Meaning of the Study

Our study showed that in general end-user satisfaction with the web-based HRA was high, but qualitative analysis of free-text field remarks indicated that there still are opportunities to increase satisfaction. We know from a previous study that satisfaction with the HRA is positively related to initiation of health behavior change after participation [16]. This suggests that by improving satisfaction based on our findings, we also extend the effectiveness of HRAs.

From our findings, confidence seems to emerge as a key construct in the satisfaction with the HRA. Also, the confusion or disagreement of the GPs about the feedback reported by participants suggested that the evaluated HRA was not optimally embedded in the health care system. Therefore, the HRA should be seamlessly intertwined into the current health care practice. First, this might increase the familiarity of health professionals with the feedback provided by the HRA, which may encourage the use of this feedback by the GP. Second, when the HRA is offered by a health professional itself (eg, GPs), confidence of end-users in the HRA and the provided feedback might increase. Offering the HRA via the GP might also increase initial participation. Colkesen et al showed that one of the most frequently mentioned reasons for not participating in the HRA was that people were already under supervision of a physician [31]. In such cases, the physician can explain why it might still be relevant for them to participate in the HRA.

For developers of HRAs, this study provides insight into implications for HRAs and potential improvements. Transparency of the provided feedback is an important aspect to consider. Furthermore, information regarding the aim and content of the HRA should be clear and accurate, to prevent unrealistic expectations among end-users. For example, end-users should know the benefits and limitations of the HRA but also need to be educated about the advantages and disadvantages of screening in general.

### Unanswered Questions and Future Research

 Our study evaluated end-user satisfaction with a web-based HRA with tailored feedback and showed that respondents with elevated health risks were less satisfied with the comprehensibility of the provided feedback. Remarks showed that these respondents had difficulties applying the lifestyle advice and reported anxiety as a result of the feedback of the HRA. As lifestyle behavior change and other health-related actions will be most needed in this group of respondents, further research is needed to provide an insight into how the feedback can be improved and into the relation between respondent’s satisfaction rankings and the actual health-related behavior. For instance, it is possible that respondents are more critical because of an increased awareness about their health. Future research should also focus on the relationship between confidence in the HRA and satisfaction rankings.

Furthermore, before and during use of an HRA, end-users will have various expectations with regard to an HRA’s output and usefulness. This will influence their willingness to use and their eventual satisfaction with the system in question. For example, another important reason for non-participation in this HRA was that many potential users felt they were healthy and therefore not in need of screening [31]. Providing accurate information tailored to this particular group of users might increase participation. Furthermore, our study showed that during use, the expectations of participants did not match with the HRA’s output, which influenced their satisfaction. Although these expectations with regard to the outcome and usefulness of a HRA might vary, it will probably be possible to distinguish clusters of (potential) users with similar expectations. If these are known, tailored HRA solutions and information about HRAs that increase participation and eventual satisfaction can be developed.

### Conclusion

In general, respondents were satisfied with the web-based HRA with tailored feedback. However, information about elevated health risks was not always received well. Our study showed that respondents with elevated health risks were less satisfied with the comprehensibility of the feedback given by the HRA and were suspicious of the feedback. Furthermore, there was a lack of confidence in the HRA, as respondents felt they could not exert control over the health information and therefore they perceived that the HRA did not reflect their health status correctly. As a result, several respondents remarked they would prefer a personal face to face consult with a health professional. This suggests a need for a more responsive and flexible approach.

An important aspect in improving the web-based HRA is to increase the transparency of the generation of the provided feedback for both health professionals as well as participants. In particular, it is necessary to improve the embedding of the HRA in current health care practice. This might result in increased use and confidence in the HRA.

## Multimedia Appendix 1

Example of a personal health risk profile and lifestyle behavior advice.

## Multimedia Appendix 2

Simplified example of the generation of the feedback provided by the HRA.

## Multimedia Appendix 3

Characteristics and interpretations of the codebook used to structure the data.
