# Supplementary material for: Evaluation of End-User Satisfaction Among Employees Participating in a Web-based Health Risk Assessment With Tailored Feedback
Source: J Med Internet Res. 2012 Oct 30;14(5):e140. doi: 10.2196/jmir.2067 (PMC3510759; doi:10.2196/jmir.2067)
Supplement: Supplementary file 1 [file jmir_v14i5e140_app1.pdf]

Multimedia Appendix 1: Example of a personal health risk profile and lifestyle behavior advice.

Respondent

Respondent: 42-year-old man, with a total moderate-intensity physical activity of 30 minutes three days a week, eats one portion of fruit and 100 grams of vegetables a day, and has a mean blood pressure of 132/85 mmHg. He is highly motivated for health improvement, is in the preparation stage for physical activity, has a low self-efficacy, and has a preference for non-guided improvement of physical activity.

Figure 1 shows a screenshot of the personal health risk profiles page of the web-based feedback for this respondent. An orange bar is shown for physical activity because he does not meet the national guidelines for physical activity. An orange bar is also shown for nutrition because his intake of fruit and vegetables is below the recommended level, according to healthy nutrition guidelines. Finally, an orange bar is shown for blood pressure, because he has a slightly elevated systolic and diastolic blood pressure, according to cardiovascular disease risk-management guidelines. The respondent can get further information about the personal health risk profile by clicking on the button next to the coloured bars. This information is tailored to his motivation and stage of change (ie, highly motivated and in the preparation stage).

Table 4 gives an example of the feedback on this respondent's physical activity profile. When the button on the bottom right-hand side is clicked, the respondent goes to the lifestyle behavior advice page of his feedback. For this respondent, the lifestyle behavior advice includes:

1. A physical activity improvement schedule to reach recommended levels of physical activity, which he can implement himself (ie, non-guided physical activity improvement).
2. A nutrition scheme for implementing daily fruit and vegetable intake.
3. Advice to schedule an appointment with the program staff to re-measure his blood pressure three months after he implements the recommended lifestyle changes.

Figure 1. Screenshot of the personal health risk profile page.

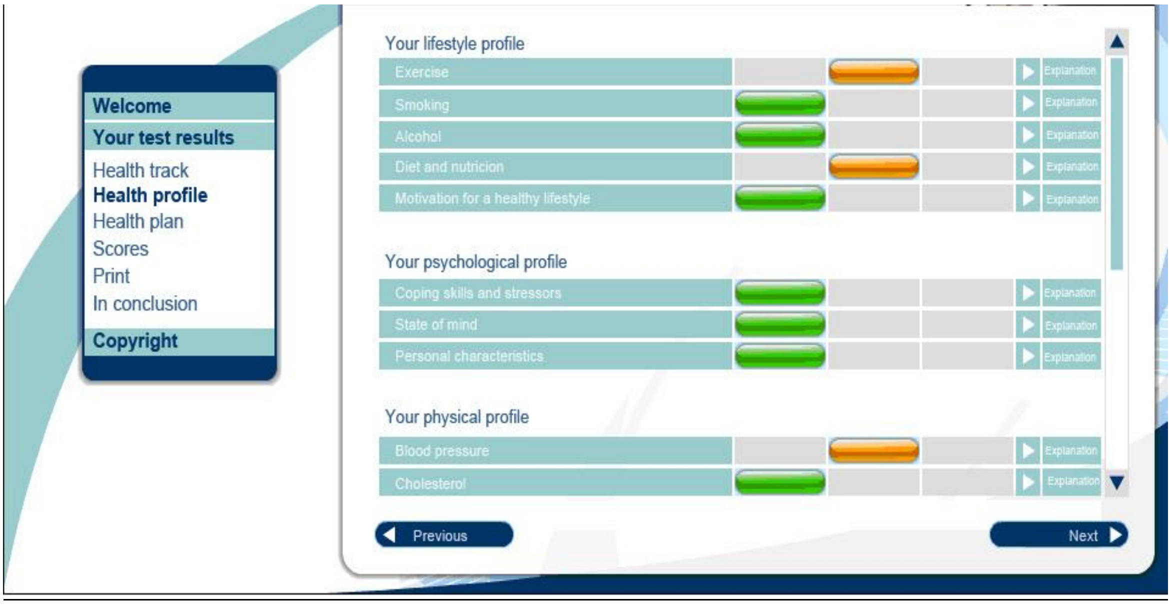

Table 4. An example of the tailored feedback on a respondent's physical activity provided by the HRA.

What does this mean for me?

Your current moderate-intensity physical activity is 30 minutes, three days a week. An activity level of less than 30 minutes of moderate-intensity physical activity, five days a week is considered insufficient.

Regular physical activity helps improve your overall health and fitness and reduces your risk for many chronic diseases (including heart attack, colorectal cancer, and diabetes) as well as improving your overall vitality and quality of life.

For you, it is also important to know that increasing your physical activity will help you control your slightly elevated blood pressure.

What can I do?

It is very positive that you already realize the benefits of regular physical activity and have been trying to make exercise a part of your life. You are not always confident that you can maintain your exercise routine. Identifying problem situations and barriers to exercise is the first step to figuring out how to overcome them.

In your lifestyle behavior advice section, you will find further tips for increasing your physical activity to recommended levels.
