# Supplementary material for: Evaluation of End-User Satisfaction Among Employees Participating in a Web-based Health Risk Assessment With Tailored Feedback
Source: J Med Internet Res. 2012 Oct 30;14(5):e140. doi: 10.2196/jmir.2067 (PMC3510759; doi:10.2196/jmir.2067)
Supplement: Supplementary file 2 [file jmir_v14i5e140_app2.pdf]

Multimedia Appendix 2: Simplified example of the generation of the feedback provided by the HRA.

After completing all HRA components, the collected information is processed by computer algorithms to provide tailored feedback. Feedback is provided with respect to five health-related domains, each of which is divided into a number of aspects (Table 5). For every aspect, it is determined whether it needs "no attention" (color green), "attention" (color orange), or "professional attention" (color red).

A compass metaphor is used to summarize the overall health risk across all aspects, using the categories "on track", "slightly off-track", "moderately off-track", and "seriously off-track". Slightly simplified, the overall health risk is determined as follows. If there are no aspects that need "professional attention" and only one aspect that needs "attention", the participant's overall health risk will be "on track". If only one aspect needs "professional attention", or up to two medical, work-related or psychological aspects need "attention", the participant's feedback will be "slightly-off track". If two or more medical or psychological aspects need "professional attention", or three or more aspects need "attention", the participant's feedback will be "moderately off-track". Otherwise the participant's feedback will be "seriously off-track".

A practical example: when the biometric evaluation of the participant shows elevated cholesterol levels and a high blood pressure that require "professional attention" and the health and lifestyle questionnaire indicates an unhealthy diet, this participant will be "slightly off-track". If the questionnaire also indicates that the participant is at risk for burn-out, the participant will be "moderately off-track".

In Figure 2, the algorithm for the cardiometabolic health risk is shown. This algorithm combines collected data from the questionnaire and the biometric evaluation. The key variable in this algorithm is SCORE, the estimated individual risk of a fatal cardiovascular event within the next 10 years [1]. This SCORE variable has its own algorithm (not shown), which combines smoking status, age, gender, total cholesterol, and systolic blood pressure.

Table 5. Health related domains and different aspects, covered by the HRA.

Risk domains included in the algorithm to determine the overall health risk
BEHAVIORAL DOMAIN
1. Physical activity
2. Smoking
3. Alcohol consumption
4. Diet
5. Motivation for health-related behavior change
6. (Self-)efficacy
7. Psychological characteristics and preferences for behavioral change
PSYCHOLOGICAL DOMAIN
8. Stress
9. Vitality
10. Burn-out
11. Depression
12. Anxiety
MEDICAL DOMAIN
13. Blood Pressure
14. Lipid profile
15. Glucose
16. Body Weight and Fat Distribution
17. Lung Function
18. Kidney Function
19. Osteoporosis
20. Vision
21. Hearing
22. Physical complaints: arm-neck-shoulder (CANS)
23. Physical complaints: back and lower extremities
PERSONAL MEDICAL HISTORY/FAMILIAR RISK DOMAIN
24. Cardiovascular disease
25. Cancer (colorectal, breast, prostate, melanoma)
26. Diabetes
WORK-RELATED DOMAIN
27. Workability
28. Work Engagement

Figure 2. Algorithm for cardiometabolic health risk

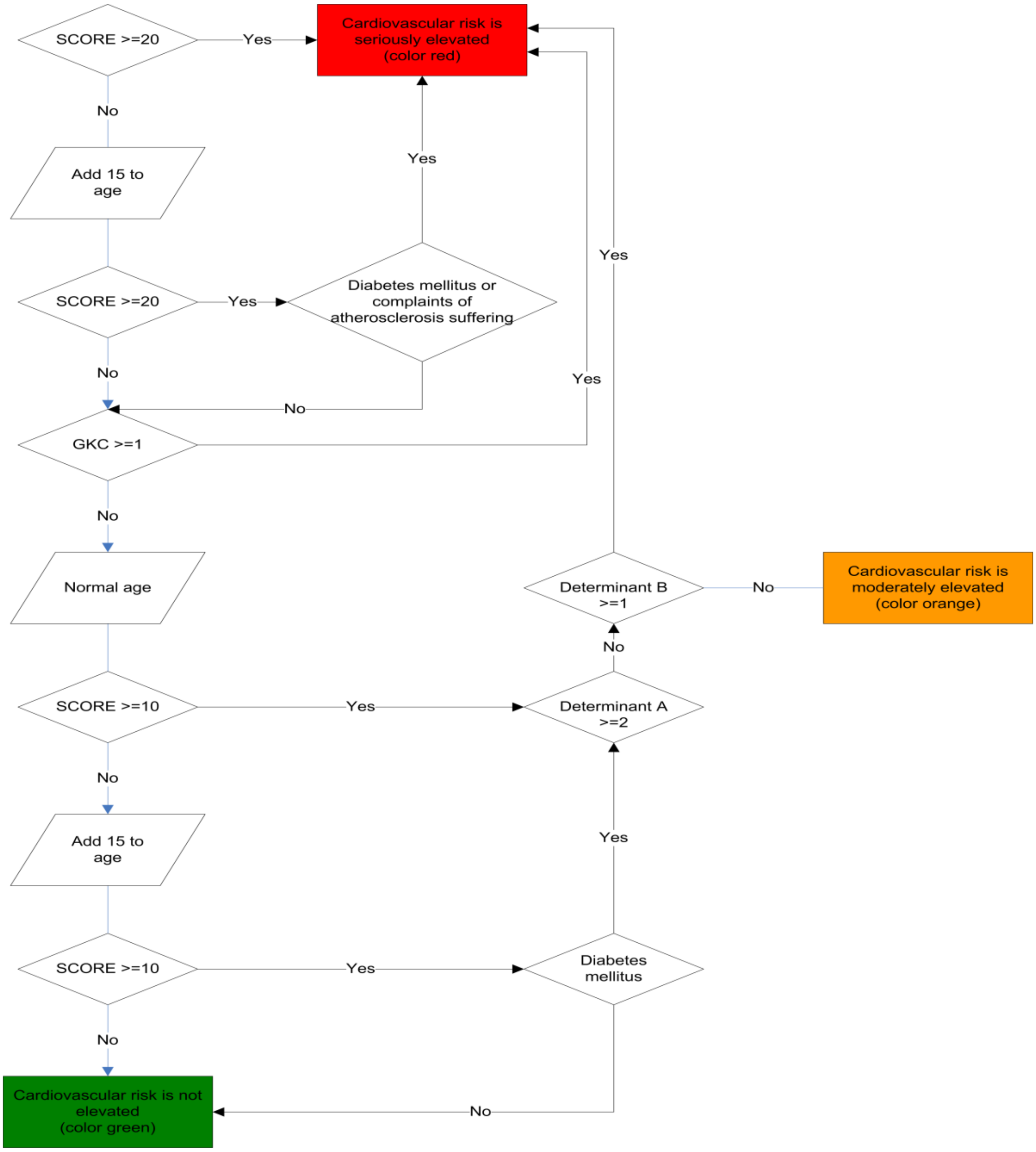

GKC = the amount of previously diagnosed cerebrovascular, peripheral vascular or cardiac diseases. Determinant type A = a family member below 65 years with heart disease OR physical inactivity OR obesity OR moderately reduced kidney function.

Determinant type B = a family member below 65 years with heart disease OR a seriously reduced kidney function OR, obesity (> 35kg/m2) OR intima-media thickness of the artery walls > 1 OR both high blood pressure and left ventricular hypertrophy.

References

1. Conroy R. Estimation of ten-year risk of fatal cardiovascular disease in Europe: the SCORE project. *European Heart Journal.* 2003;24:987-1003.
