# Supplementary material for: Evaluation of End-User Satisfaction Among Employees Participating in a Web-based Health Risk Assessment With Tailored Feedback
Source: J Med Internet Res. 2012 Oct 30;14(5):e140. doi: 10.2196/jmir.2067 (PMC3510759; doi:10.2196/jmir.2067)
Supplement: Supplementary file 3 [file jmir_v14i5e140_app3.pdf]

Multimedia Appendix 3: Characteristics and interpretations of the codebook used to analyze the data.

The codebook is based on Wixom and Todd, 2005 (Table 1, p.88). The table describes various domains (Wixom and Todd use the term "external variables") divided into various concepts (Wixom and Todd use the term "instrument characteristics"). Every remark was first coded with a domain, after which an appropriate concept was added. If the prevailing concept did not exist within the domain, concepts were copied from other domains or an appropriate concept was added to the codebook. Definitions are adapted from Wixom and Todd, 2005; and Bailey and Pearson, 1983. Definitions were specified further to the HRA in the current study and adjusted during the qualitative analysis.

Table 6. Characteristics and interpretations of the codebook.

**System quality**	Users' perceived quality of the web-based component of the HRA.	
	Accessibility	*The degree to which the system is accessible to its users.*
	Timeliness	*The availability of system's output at a time suitable for its use.*
	Flexibility	*The capacity of the system to change or adapt in response to new conditions, demands, or circumstances.*
	Integration	*The ability of the system to communicate/transmit data between its different functional areas.*
	Efficiency	*The rate or speed at which the system enables users to accurately and successfully complete a task.*
	Errors[a]	*The methods and policies governing correction and rerun of incorrect system output.*
	Tailoring[b]	*The ability of the system to take user characteristics into account.*
**Information quality**	Users' perceived quality of the information given by the HRA.	
	Accuracy	*Users' perception that the information is correct.*
	Precision	*The variability of the output information from that which it purports to measure.*
	Completeness	*The degree to which the HRA provides all information perceived as necessary by the user.*
	Format	*The layout and display of the information throughout the entire web portal.*
	Volume	*The amount of information conveyed to users.*
	Control[a]	*Users' perceived power to regulate/influence the information that is entered into or provided by the system.*
	Language[b]	*Users' ability to understand the language used in the HRA.*
**Service quality**	Users' perceived quality of the service delivered by professionals associated with the HRA.	
	Relationship with the staff that is associated with the HRA	*The method and manner of interaction between users and staff.*
	Communication with the staff that is associated with the HRA	*The way information is exchanged among users and staff.*

	Technical competence of the staff that is associated with the HRA	*The skills and expertise of the staff.*
	Schedule of products or services	*The timetable for system output, services, and procedures.*
	Processing of change requests	*The manner, method, and required time the staff respond to users' requests.*
	Vendor support	*The type and quality of services delivered by external parties (eg, the measurement instruments at the location for biometric evaluation).*
	Response time	*The time between users' requests for service or action and response to these requests.*
	Means of input of the HRA	*The method and medium by which users receive services from staff and/or the system and the perceived usefulness of this service.*
	Staff support[b]	*The service and help offered by the staff associated with the HRA.*
**Usefulness**	General usefulness of the HRA for its users.	
	Usefulness	*The extent to which the HRA actually helps to solve users' problems.*
	Relevancy	*The degree of congruence between users' needs and requirements and what the HRA provides.*
**Ease of use**	Degree to which users believe that using the HRA is effortless.	
	User-friendly	*The HRA is pleasant to use and easy to learn.*
	Easy to use	*The HRA effectively fills users' needs and is fast and free of errors.*
**Outcome expectations**	Congruence between users' expectations and actual situation with regard to using the HRA and the feedback provided by the system.	
	Expectations	*Users' expectations of the HRA.*
	Confidence in the system	*Users' feelings about the reliability of the HRA and the feedback provided by the system.*
	Feelings of control	*Users' perceived power to regulate/influence the feedback provided by the system.*
	Health effects	*Changes in lifestyle or other health-related issues as a result of using the HRA.*
	Accuracy[a]	*Users' perception that the provided feedback is congruent with their expectations about their health.*
	Tailoring[b]	*Congruence between users' expectations after completing successive parts of the HRA and the actual situation.*
**Organizational factors**	Influence of the organization, procedures, and choices on the quality of the HRA.	
	Organizational competition	*Congruence between the assessments and feedback provided by the system and an external health professional.*
	Error recovery	*The way the staff and organization manage errors as a consequence of the service delivered by the company behind the HRA.*
	Data security	*The safeguarding of data from misappropriation or unauthorized access, alteration, or loss.*
	Communication[a]	*The availability of correct information before using the HRA.*
	Time[a]	*The availability of the evaluation questionnaire at a time suitable for its use.*

[a] Concept is copied from another domain.

[b] Concept is added to the codebook.

Note: Concepts that were part of the original codebook but not used during analysis are not described in the above table. These characteristics were language (system quality), reliability, currency (information quality), attitude of staff associated with the HRA, time required for new development, processing of change requests (service quality), understanding of systems, feelings of participation, degree of training (outcome expectations), priorities determination, involvement of top management, charge-back method, documentation, and the organizational position of staff (organizational factors).
